# Transcriptome Analysis of Canola (*Brassica napus*) under Salt Stress at the Germination Stage

**DOI:** 10.1371/journal.pone.0116217

**Published:** 2015-02-13

**Authors:** Weihua Long, Xiling Zou, Xuekun Zhang

**Affiliations:** Key Lab of Biology and Genetic Improvement of Oil Crops, Ministry of Agriculture, Oil Crops Research Institute of Chinese Academy of Agriculture Science, Wuhan 430062, China; University of Delhi South Campus, INDIA

## Abstract

Canola (*Brassica napus*) is one of the most important oil crops in the world. However, its yield has been constrained by salt stress. In this study, transcriptome profiles were explored using Digital Gene Expression (DGE) at 0, 3, 12 and 24 hours after H2O (control) and NaCl treatments on *B. napus* roots at the germination stage. Comparisons of gene-expression between the control and the treatment were conducted after tag-mapping to the sequenced *Brassica rapa* genome. The differentially expressed genes during the time course of salt stress were focused on, and 163 genes were identified to be differentially expressed at all the time points. Gene Ontology and Kyoto Encyclopedia of Genes and Genomes enrichment analyses revealed that some of the genes were involved in proline metabolism, inositol metabolism, carbohydrate metabolic processes and oxidation-reduction processes and may play vital roles in the salt-stress response at the germination stage. Thus, this study provides new candidate salt stress responding genes, which may function in novel putative nodes in the molecular pathways of salt stress resistance.

## Introduction

Salinization of agricultural land has been a major problem worldwide for decades. According to Food and Agriculture Organization (FAO land and plant nutrition management service, 2008), 7% of the world’s land has been salinized, and this percentage is still increasing. As one of the major abiotic stresses, salt stress greatly reduces crop productivity. Meanwhile, the worldwide population is predicted to grow to 9 billion by the end of 2050 (www.un.org/popin/data.html.). Thus, understanding plant-salt interaction mechanisms and breeding salt-tolerant crops will be invaluable to secure the world’s food supply [1–3].

High soil salt level can result in physiological drought and ionic poisoning in plants by decreasing their water potential and causing the accumulation of excess ions [4]. Plants evolve several mechanisms to cope with salt stress, such as ion homeostasis, osmotic homeostasis, redox equilibrium, growth regulation and others [5]. These mechanisms are all achieved through corresponding physiological and biochemical changes by regulation of numerous salt-responsive genes. One such group of genes consists of structural protein-coding genes, including osmoregulatory genes, antioxidant proteins, late embryogenesis abundant (LEA) proteins, and transporters/antiporters. For example, Ren *et al*. (2005) cloned a major quantitative trait locus encoding a high-affinity K^+^ transporter (HKT)-type sodium transporter that recycles the Na^+^ from the shoots to the roots [6]. Another group of salt-response genes includes transcription factors (TFs), such as ERF and WRKY, and signal-related protein kinases [7]. By combining the interaction mode among the mentioned genes above, several pathways that potentiate the salt-stress signal have been revealed, such as the salt overly sensitive (SOS) pathway [8–9], the calcium-dependent protein kinase (CDPK) pathway [10] and the mitogen-activated protein kinase (MAPK) pathway [11]. Furthermore, plant hormones, such as abscisic acid (ABA), ethylene, salicylic acid, and jasmonic acid, also play important roles in stress signaling and adaptation [12–14]. In spite of the progress that has been made in detailing these processes, the integrated mechanisms of salt tolerance in plants require further exploration.

Canola (*Brassica napus*) is one of the most widely cultivated oil crops in the world because of the healthy fatty acid composition of its oil and high protein content of its meal. Similar to the other crops, *B. napus* is threatened by salt stress, especially in arid and semi-arid countries (or regions). Its salt tolerance mechanism at the molecular level remains unclear, despite of a few recent reports [15–17].

Next generation sequencing (NGS), with the advantage of its cost-effectiveness and high-throughput characteristics, is the most promising method to explore molecular profiles for non-model crops [18]. And it has been applied successfully to detect transcriptome variation in *B. napus* in at least three applications to date. Firstly, genes that are expressed in distinct species, organs or conditions have been identified [19–21]. Secondly, single nucleotide polymorphisms in transcript sequences have been analyzed, which made associative transcriptomics more efficient for delineating regions of the genome that control traits and provide markers [22, 23]. Thirdly, conserved and novel microRNA functions in specific organs have been explored [24]. Digital Gene Expression (DGE) profiling, based on an NGS platform, has been applied to gene-expression comparisons of different species, developmental stages and stresses [25].

To date, there has been little transcriptome information in response to salt stress in *B. napus*. The root is the first mediation organ exposed to salt stress; therefore, it may have primary functions in the interaction process. In this study, we applied DGE profiling to root samples at 0-, 3-, 12- and 24-hour time points under control and salt treatments, to explore the transcriptome profiles and to identify salt-responsive genes of *B. napus* at the germination stage.

## Materials and Methods

### 2.1. Plant material and stress treatment

Healthy seeds of *B. napus* line WH126 were surface-sterilized with 5% sodium hypochlorite for 5 min and washed three times with ddH_2_O. About 700 clean seeds were placed on eight-layer filter papers supplied with adequate water (dH_2_O) in petri dishes (diameter = 13 cm) in a climate chamber (25 ± 1°C, 130 *μ*mol/m^2^ s in light and 60–80% in humidity). When young radicles emerged (approximately 1–2 mm in length out of the seed coats), 600 uniform seeds were distributed into six dishes, three of which were treated with dH_2_O (control) and the others with 1.25% NaCl solution (salt treatment, abbreviated as ST). The three dishes of each treatment were thought of as three replicates. The subsequent culture procedures were the same for each treatment. The chosen NaCl concentration was based on our previous research [26]. Four time points (0-, 3-, 12- and 24-hour) after transfer of the seedlings were set as sampling nodes. At each time point, whole roots under control or ST were cut as samples (named H0, H3, H12, H24 and S3, S12, S24; where H and S correspond to dH_2_O and ST, respectively) and flash frozen in liquid N_2_ for storage. Note that the H0 represented the starting sample for both treatments. Three biological replications were prepared and the corresponding root samples from each replication were mixed equally for subsequent steps.

### 2.2. Measurement of root length

At the sampling points of each replicate, 10 typical germinated seeds from each dish under control and ST conditions were photographed. The root lengths of each treatment at each time point were calculated using the software Image J, by referring to the graph paper scale plate. Software Origin 8.5.1 was used to draw the figure using the data from Image J.

### 2.3. RNA extraction and library construction

RNA was extracted from seven mixed root tissues (H0, H3, H12, H24, S3, S12 and S24) following the protocol of Total RNA Purification Kit (TRK1001, LC Science, Houston, TX, USA). The quality and purity of the RNA were evaluated by Bioanalyzer 2100 and RNA 6000 Nano Lab Chip Kit (Agilent, Santa Clara, CA, USA).

Fifty nanograms of purified RNAs were taken from each RNA sample to construct the libraries, and the rest of the RNA was carefully preserved for later use. Following the detailed protocol of the Gene Expression Sample Prep Kit (Illumina, San Diego, CA, USA), including the important steps of mRNA purification, mRNA fragmentation, adding adapter, reverse transcription and library validating, seven corresponding gene-expression libraries were successfully constructed, named H0, H3, H12, H24, S3, S12 and S24. These libraries were deep sequenced on an Illumina Hiseq2500 at the LC-Bio Company (Hangzhou, China). The generated image files were processed to produce digital sequence data.

### 2.4. Sequence analysis

Through base calling, the 36-bp nucleotide sequences originated from the Illumina platform were designated as raw data. The raw data were filtered by removing the following four types of sequence tags: (1) ≥ 5-bp dimer-containing tags, (2) ≥ 3 bp of ambiguous nucleotides, (3) 3’-adapter tags and (4) tags with sequence quality values < Q20. The remaining sequences were considered as clean tags (the corresponding data have been submitted to the SRA database of NCBI with the ID PRJNA237675).

The clean-tag sequences of each library were mapped to the *B. rapa* transcripts (ftp://ftp.jgi-psf.org/pub/compgen/phytozome/v9.0/Brapa/annotation/Brapa_197_transcript.fa.gz) using Bowtie software to monitor and count the mapping events on both the sense and the complementary antisense sequences from the transcript database. Only a one-nucleotide mismatch was permitted in this process.

The fragments per kilobase of transcript per million mapped reads (FPKM) method was used to map the clean data for each gene, which indicated the gene-expression level. In this experiment, a *p* value ≤ 0.05 and a |log2FPKM ratio| ≥1 were set as the thresholds to determine the significance of gene-expression difference between samples. Notably, gene-expression comparisons of the samples at the 3-, 12- and 24-hour time points (S3/H3, S12/H12 and S24/H24) were performed. The Mev software produced heat-maps representing the expression patterns of the Differentially Expressed Genes (DEGs) and gene transcripts at each time point.

Gene Ontology (GO) and Kyoto Encyclopedia of Genes and Genomes (KEGG) information for the DEGs were obtained from Phytozome v9.1 BioMart annotation with the selected organism as *B. rapa* (http://www.phytozome.net/). For functional enrichment analysis of the DEGs, their GO terms were compared with the genome background and the derived *p* values were calculated according to Wang *et al*. [27]. Similarly, the pathway enrichment analysis was done using the KEGG database. For each of these analyses, a *p* value <0.05 was required for differences to be considered statistically significant.

### 2.5. Quantitative real-time PCR (qPCR)

Three biological replications with two technique replications of RNA were used for qPCR analysis. After being treated with RNase-free DNase, RNA samples were used as templates for reverse transcription with the M-MLV RTase cDNA Synthesis Kit (Takara, Dalian, China). Primers were designed using PRIMER3 software and listed in S1 Table. The expression of *β*-actin gene was used as a control. About 1 μl of the cDNAs of each sample were used for ordinary PCR to test whether the corresponding primers generated products. Real time PCR was carried out with the SYBR Green PCR Master Mix system (Takara) on an ABI 7500 Real-time PCR platform. The PCR amplification conditions were performed according to Zou *et al* [21]. The ΔΔC_t_ Value for each gene was analyzed in Microsoft Excel and used to indicate the change in expression level between two samples.

## Results

### 3.1. Effects of salt stress on *B. napus* root development

**Fig 1:**
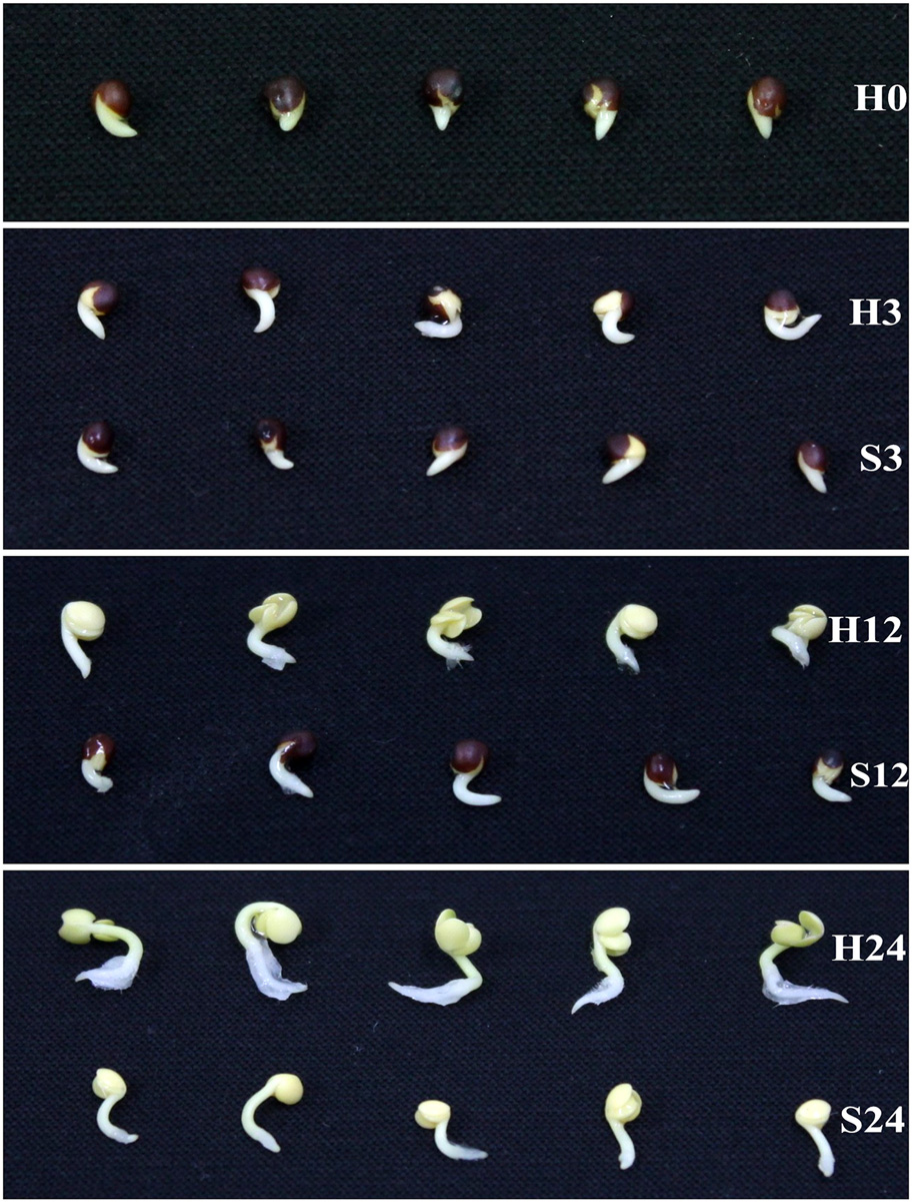
Phenotypes of germinated seeds of *Brassica napus* line WH126 at 0, 3, 12 and 24 hours after H_2_O (control) and salt (1.25% NaCl) treatments. The names on the figure refer to the corresponding rows of germinated seeds. H indicates H_2_O treatment, S indicates salt treatment and the adjacent numbers indicate the sampling time points. The names were also used to describe the corresponding cDNA libraries.

**Fig 2:**
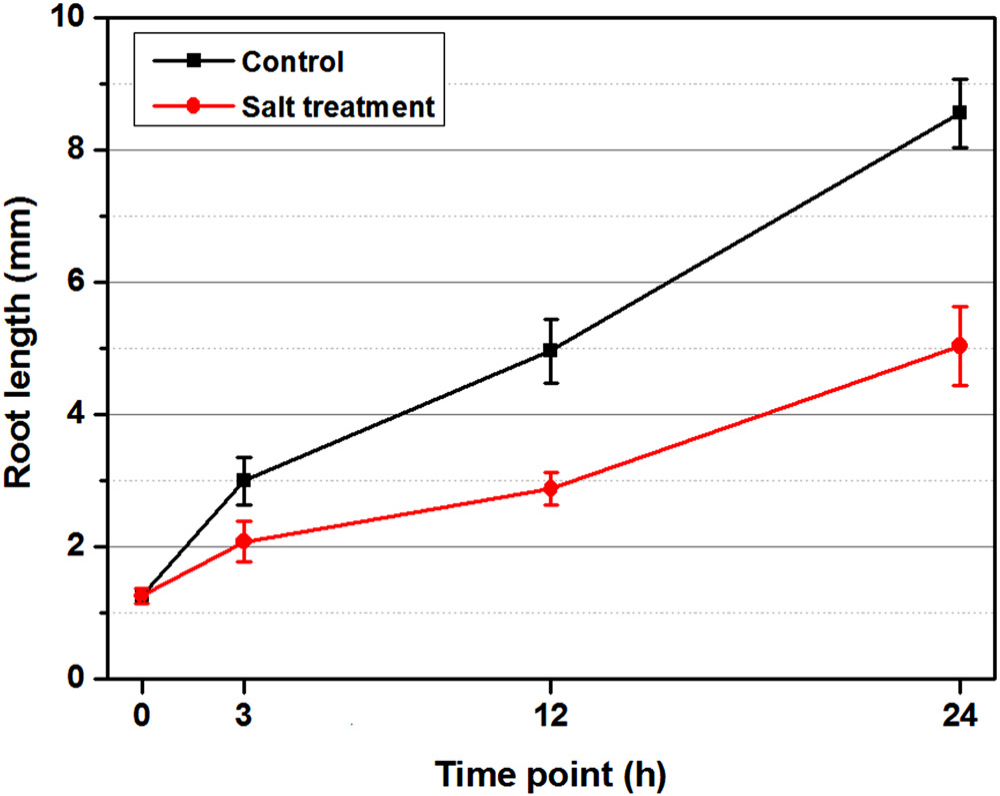
Effect of salt (NaCl) on *Brassica napus* roots length during the 24-hour time course. Equal numbers of seeds were placed in three petri dishes under control (H_2_O treatment) and salt treatment (ST), separately, and placed randomly in the incubator. At each time point, the uniform germinated seeds under both treatments were sampled from every dish to capture root-length values. The whole experiment was carried out on three replicates. The error bars represent the standard error (SE) of the mean root length.

Seeds should experience a different process of water uptake under the control and salt treatment (ST) during imbibition. To avoid this complication, the germinated seeds were used in this study (H0 in Fig. 1). Changes in root length under two treatments were shown in Fig. 2. In the first 3 hours, uniform vigorous seeds developed with no obvious visible differences between the control and ST. However, the roots under the control were longer than those under ST at 12 hours, and this tendency was more evident in the following periods. The cotyledons of seeds under the control broke out of the coats completely at 12 hours, and those under ST emerged at 24 hours. In addition, roots were with root hairs under the control at 24 hours, which was not seen under ST.

### 3.2. Illumina sequencing and data analysis

Root samples from *B. napus* line WH126 at four time points (0, 3, 12 and 24 hours) under ST and control conditions were used to construct 7 libraries for sequencing (Table 1). We obtained 8 million to 24 million raw reads from each of the seven libraries. After the process of adaptor deletion, junk filtering and low copy filtering, >99% of the sequences were confirmed as clean data. Through mapping to the *B. rapa* genome database, 54.85% to 65.61% of the clean data could be mapped, and 32,941 to 35,819 genes (>80% of the total 41,049 genes in the *B. rapa* genome) were identified. Saturation analysis revealed that the mapped gene numbers appeared stable when the data reached 4 million reads (S1 Fig.). From Table 1, for example, the raw data for the S12 library comprised 23 million clean reads, but the number of mapped genes (35,819) was almost equal to that of the S24 library, with 9 million reads. Therefore, the sequence data were sufficient to proceed with gene-expression analysis.

**Table 1:** DGE sequencing statistics.

Summary	Illumina Tag Library							
	Items	H0	H3	H12	H24	S3	S12	S24
Raw data	total	8,432,911	15,078,553	9,384,024	8,085,785	24,347,894	23,763,501	9,541,163
Raw data	unique reads	5,488,199	8,778,179	5,830,925	5,384,214	13,904,391	12,951,510	6,227,440
Clean data	total	8,395,637	15,006,145	9,336,180	8,037,374	24,240,652	23,659,776	9,481,273
Clean data	unique reads	5,458,027	8,721,040	5,791,613	5,342,824	13,821,175	12,873,390	6,178,792
All data mapping to gene	total number	5,508,516	9,159,581	5,493,082	4,408,534	13,985,378	14,486,393	5,786,340
All data mapping to gene	% of total clean reads	65.61%	61.04%	58.84%	54.85%	57.69%	61.23%	61.03%
All data mapping to gene	unique reads	3,599,320	5,409,508	3,468,335	3,181,089	7,974,423	7,969,495	3,789,067
All data mapping to gene	% of unique clean reads	65.95%	62.03%	59.89%	59.54%	57.70%	61.91%	61.32%
All read-mapped genes	number	32,941	34,536	34,530	34,338	35,189	35,819	35,036
All read-mapped genes	% of ref genes	80.31%	84.20%	84.18%	83.71%	85.79%	87.32%	85.41%

### 3.3. General gene-expression description during the time course of salt stress

**Fig 3:**
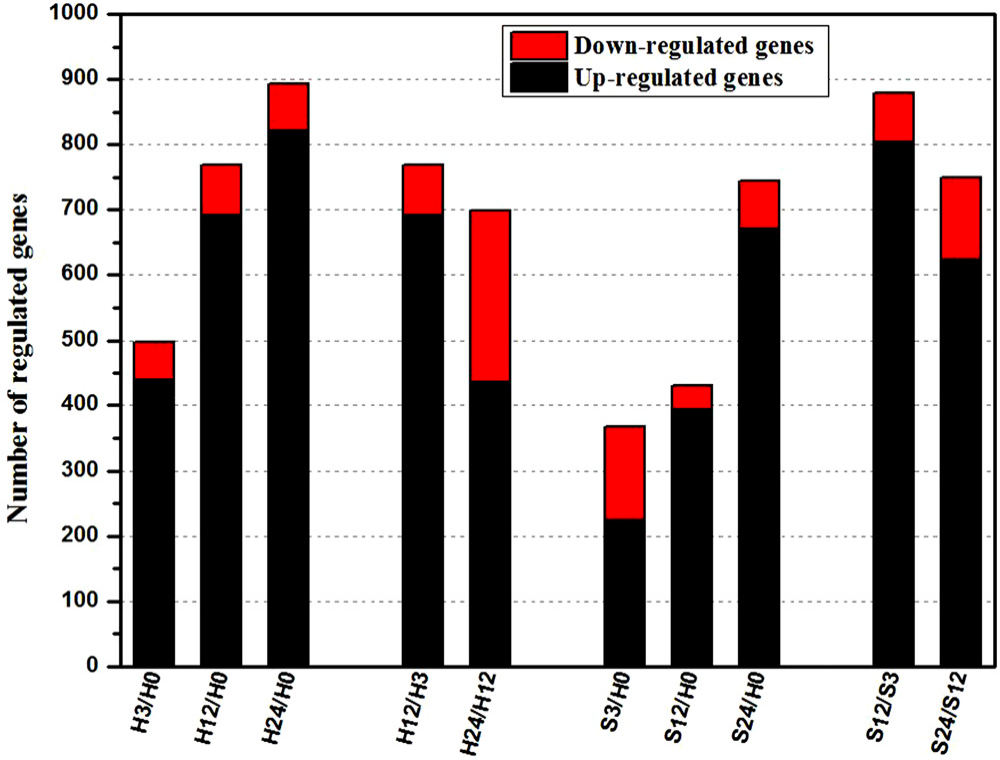
Summaries of differentially regulated genes during the time course. Differentially regulated genes were identified either by a comparison of adjacent stages or comparing each stage to H0. “H3/H0” indicates a comparison between the gene expression in the H3 library with that in the H0 library, and the same describes the other labels on the x-axis.

Compared with their expressions at H0, there were 497, 769 and 894 DEGs at the time points of 3-, 12- and 24-hour our during the control procedure, displaying a rising tendency in the numbers of DEGs. A similar performance was observed in the whole salt treatment procedure (Fig. 3). In addition, the gene-expression comparisons of adjacent time points of the two treatments also showed the significant expression change. More than 700 genes were confirmed as DEGs between two time points under the same treatment. Interestingly, the up-regulated genes outnumbered the down-regulated genes. To identify candidate genes that respond to salt stress in *B. napus* roots, we focused on the DEGs identified by comparing the gene expression levels under ST *vs*. control conditions at the same time points.

**Fig 4:**
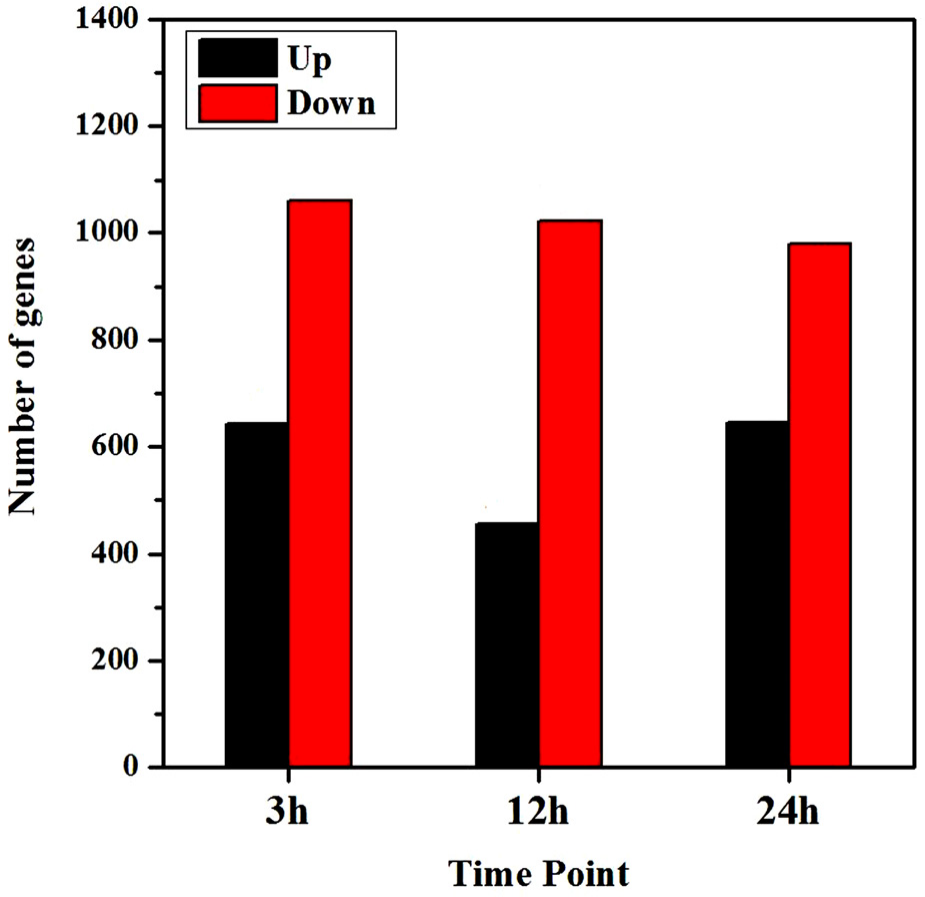
Total regulated genes at each time point after salt treatment. Differentially expressed genes were confirmed based on whether the |log_2_Foldchange| ≥1 and *p* value ≤0.05.

Of the mapped genes, 1,704 (4.93% of 34,536 genes), 1,477 (4.28% of 34,530 genes) and 1,626 (4.74% of 34,338 genes) genes were differentially expressed at 3-, 12- and 24-hour, respectively, among which 644, 455 and 646 genes were up-regulated and 1060, 1022 and 980 genes were down-regulated (Fig. 4). Down-regulated genes were much more than the numbers of up-regulated ones at each time point.

**Fig 5:**
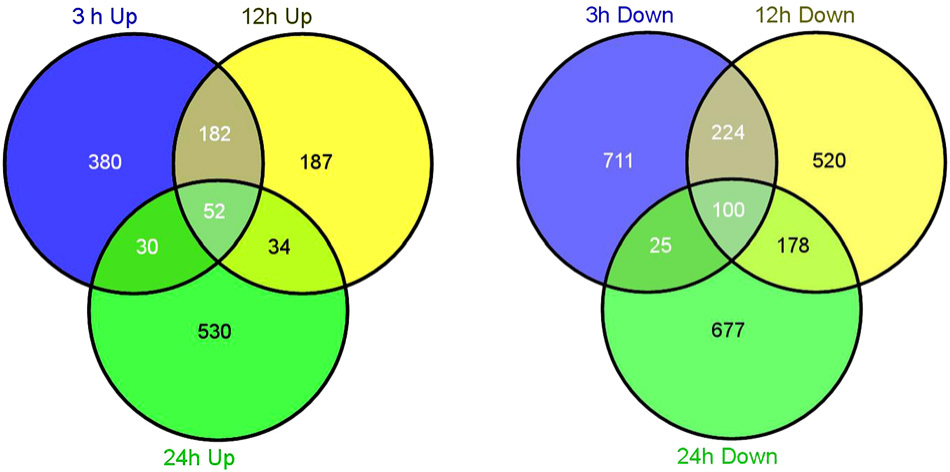
Venn diagram describing the exclusion and overlap of regulated genes at three time points. The left and right diagrams indicate the up- and down-regulated gene numbers, respectively.

The Venn diagram demonstrates the number of unique up- and down-regulated genes at each time point (Fig. 5). Most DEGs were uniquely associated with a specific time point. For example, there were 644 up-regulated genes at the 3-hour time point, and more than a half (380/644, 59%) were identified to be increased only at the 3-hour. By analyzing the DEGs during the entire treatment course, only 163 genes were expressed differentially under salt treatment at all-time points (S2 Table).

### 3.4. Expression patterns of 163 candidate genes

**Fig 6:**
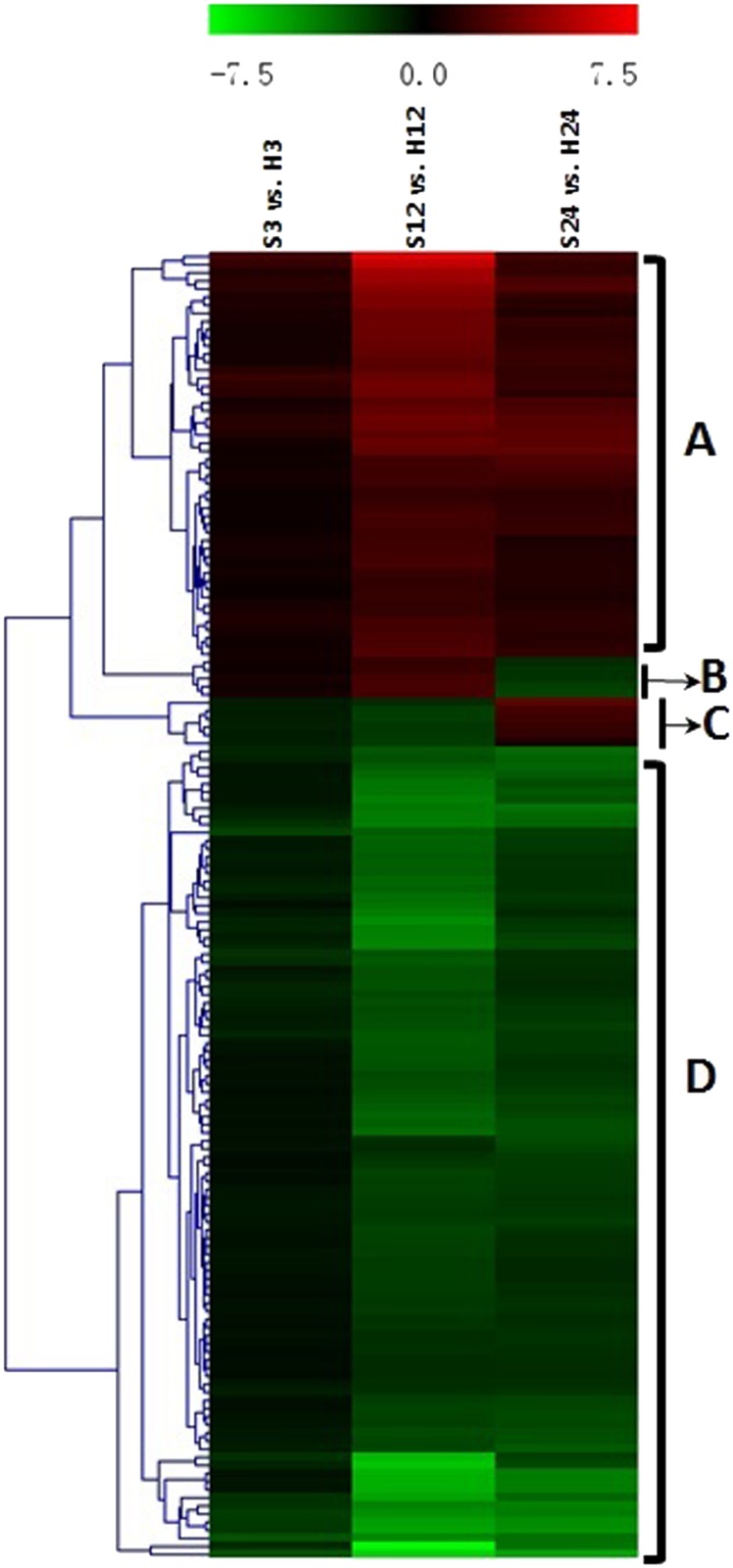
Hierarchical cluster analyses of the 163 common differentially expressed genes (DEGs) at three time points. This map shows the changes of log_2_(foldchange) values at the indicated times (3 hours, 12 hours and 24 hours) during salt stress. The genes were assigned into four clusters.

By comparing the gene expression levels between the ST and control treatments at the same time point, four dynamic expression patterns were indicated by the heat-map (Fig. 6). Cluster A (52 of 163 genes, all up-regulated) and cluster D (100 of 163 genes, all down-regulated) represented the major regulation trends under ST and contained the majority genes. Meanwhile, cluster B and C had 11 genes that changed their regulation direction during the treatment course. In cluster B, 5 genes were up-regulated at 3- and 12-hour, but down-regulated at 24-hour. In cluster C, 6 genes were down-regulated at 3-hour and 12-hour but up-regulated at 24-hour.

### 3.6. GO and KEGG analyses of salt stress-related DEGs

**Fig 7:**
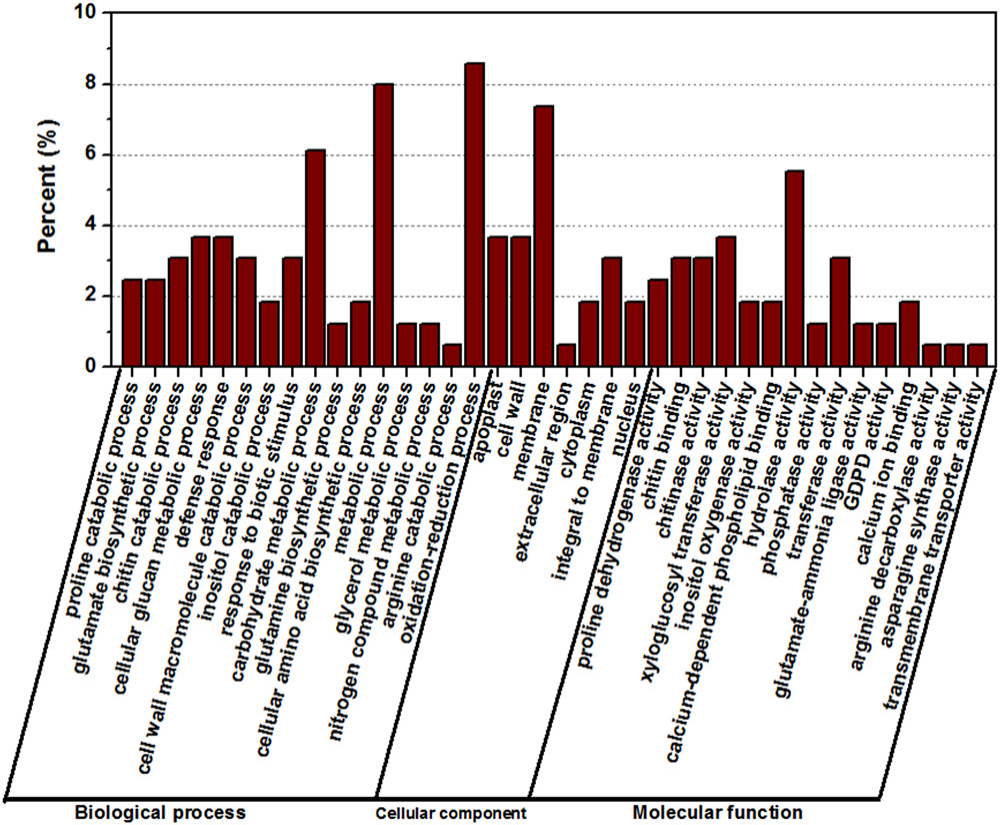
Gene Ontology (GO) analyses of commonly differentially expressed genes at three time points. The differentially expressed genes were assigned into three groups, including biological process, cellular components and molecular function. The x-axis represents the most abundant categories of each group, and the y-axis represents the percentages of the total genes in each category.

GO assignments were used to classify the functions of the 163 DEGs responding to salt stress (Fig. 7). Three non-mutually exclusive GO categories, biological process (BP), cellular component (CC), and molecular function (MF), were well represented. A total of 115 out of the 163 genes (70.55%) received GO function annotations. The most represented GO terms were presented in Fig. 7 (according to their *p*-values). In the BP category, the most abundant GO term were “oxidation-reduction process” and “metabolic process,” followed by “carbohydrate metabolic process,” “cellular glucan metabolic process,” and “defense response”. In the CC category, “membrane” was the most abundant, followed by “cell wall” and “apoplast”. Similarly in the MF category, “hydrolase activity” was the most abundant category, followed by “xyloglucosyl transferase activity,” “chitin binding,” “chitinase activity” and “transferase activity”. Notably, some genes were assigned to more than one category.

A total of 26 genes could be aligned to the KEGG pathways (S2 Table). The pathways with more mapped genes were the ascorbate and aldarate metabolism (00053); inositol phosphate metabolism (00562); alanine, aspartate and glutamate metabolism (00250); and arginine and proline metabolism (00330).

### 3.6. Identification of genes responding to salt stress

**Fig 8:**
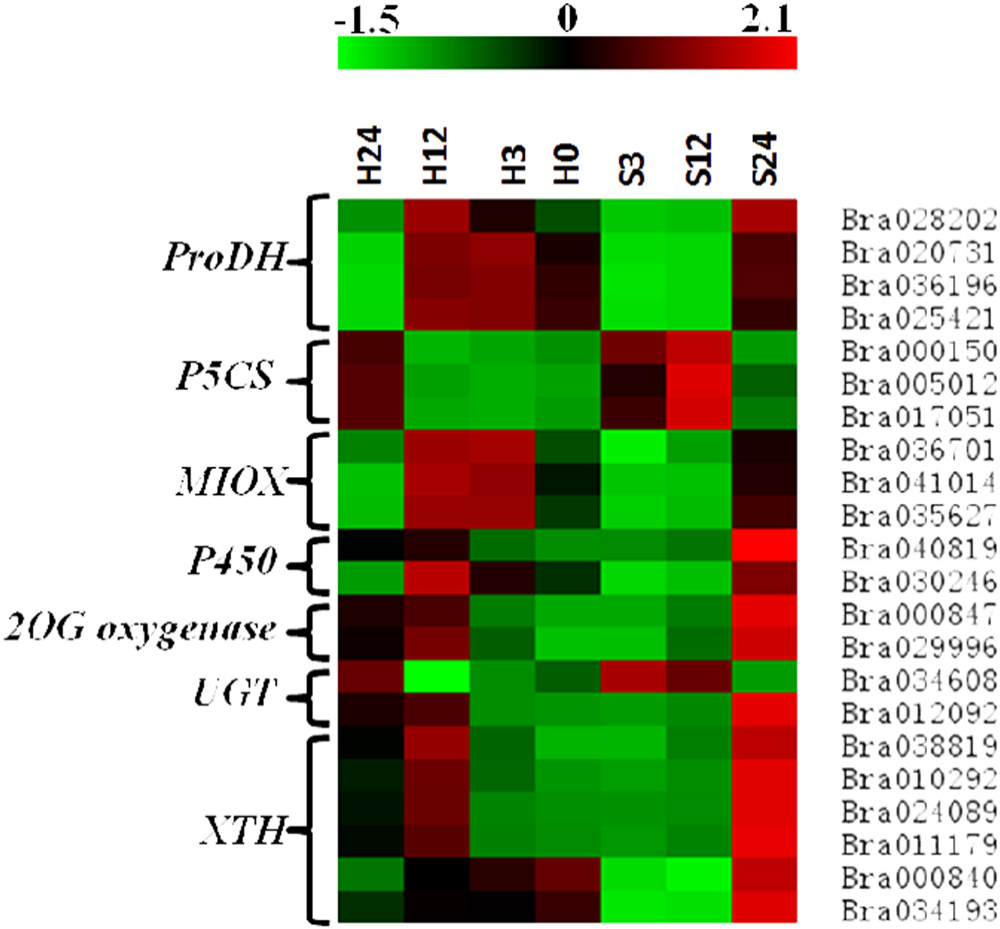
Heat-map representation for the regulation by salt stress of the focused genes. The transcript abundance of each gene at each time point under control or ST was normalized. The top color bar represents the comparative expression level. A redder color indicates more transcript accumulation, and greener indicates less.

Most genes (115/163) had Go annotations and only a few genes (26/163) had KEGG associations; therefore, we screened the salt-responsive genes according to their GO annotations. The oxidation-reduction process, metabolic process and carbohydrate metabolic process were the most significant processes (Fig. 7 and S2 Table). Therefore, these above genes were identified as the putative functional genes in related biological processes. These genes encoded enzymes including the proline dehydrogenase (ProDH), Δ^1^-pyrroline-5-carboxylate synthase (P5CS), myo-inositol oxygenase (MIOX), cytochrome P450, 2-oxoglutarate (2OG) and Fe(II)-dependent oxygenase (2OG oxygenase), xyloglucan endotransglucosylase/hydrolase (XTH). The accumulations of the transcripts of these genes at the four time points under both treatments were shown in Fig. 8. Moreover, some of these genes were among the top 10 differentially expressed genes at one or more time points under salt stress (S2 Table), indicating their important roles in salt stress resistance.

### 3. 7. Confirmation of DGE profiles by qPCR analysis

**Fig 9:**
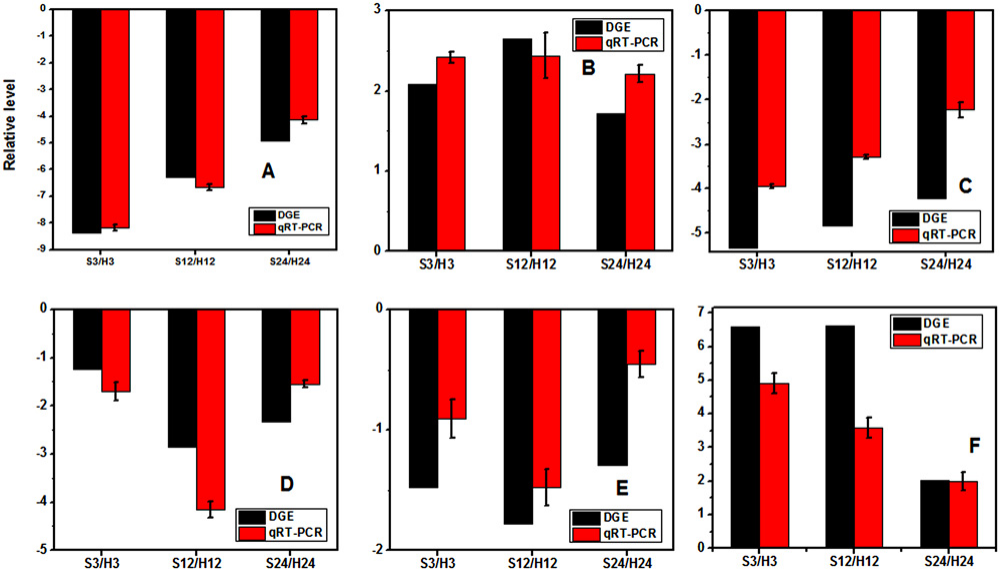
Quantitative real-time PCR (qPCR) validations of gene expression levels from digital gene expression (DGE) analysis. The qPCR values are presented as the averages of three independent experiments. The genes were randomly selected. “S3/H3” indicates a comparison between the expressions of the corresponding genes in the S3 library with that in the H3 library. The “S12/H12” and “S24/H24” ratios indicate analogous comparisons. The y-axis indicates the fold-changes obtained by two methods. The “A-F” indicates the genes with the assay names of Bra041014, Bra000150, Bra020731, Bra011843, Bra023573 and Bra000090, respectively.

To validate the results of DGE, transcriptional levels of 31 genes were evaluated by qPCR. The data of qPCR were in S3 Table. The comparisons of the relative expression levels of six genes under the two treatments were shown in Fig. 9. Although the change folds were not exactly the same as those revealed by the transcriptome profiling data, all the validated genes showed similar expression patterns that were consisted with the DGE data.

## Discussion

### 4.1. The choice of time points for transcriptome analysis

In this study, we identified important candidate genes by comprehensive investigation of the time course (within 24 hours) of the transcriptomic response to salt stress. Before the sampling of RNA-seq, a phenotype evaluation was performed. As shown in Fig. 2, root-length comparisons between the three time points demonstrated that salt stress seriously constrained root development of *B. napus*. The root lengths under control or ST had no detectable difference within the first 3 hours, but the phenotype difference became obvious with increasing time (Fig. 1). This phenomenon showed that: (1) salt stress inhibited root growth; and (2) salt stress required some time to exert its negative function on plant development. A similar phenotype was reported in Olive, *Leymus chinensis* and tomato under salt stress [28–30].

According to some studies in *Brassica rapa* [22], tomato [30] and rice [31] under ST, changes of transcriptome occur before obvious phenotypic variation. Therefore, the 3-hour time point of salt treatment is a good choice to study responding genes at the early stage under salt stress. Furthermore, Fig. 1 showed the general developmental process of germinated seeds under two treatments. The obvious phenotypic differences of germinated seeds between the control and ST were seen at 12- and 24-hour. These two time points could be the late stage to study salt-responding genes. And moreover, the time points of 0-, 3-, 12- and 24-hour were also used in previous reports [31–33]. Therefore, we chose these time points as the sampling nodes.

### 4.2 The reliability of the DGE data

There was no genome sequence for the amphidiploid of *B. napus*, which made an obstacle for genes annotation. Fortunately, the genome of *B. rapa*, one parent of *B. napus*, was released [34]. Genes of *B. napus* showed a high degree of collinearity to *B. rapa* [35, 36]. Moreover, the majority of *B. napus* sequence tags derive from transcripts with the same function as their *B. rapa* homologs [37]. Therefore, annotations of genes from *B. napus* based on *B. rapa* genome were acceptable.

Though there were no technical replicates for the DGE sequencing in this study, we believe that the results were reliable for the following reasons: (1) the expression levels of the genes from the sequence data were verified by qPCR, indicating that the outcomes derived from DGE sequence reflected the real situation of the global gene expression profiles. (2) Only the common DEGs during the time course were taken forward for analysis. These DEGs were detected at all three time points. In other words, they were captured three times during the time course, proving they responded to salt stress. (3) In contrast to the experimental design used during the time course of stress in other publications [28, 38, 39], this study set a control at every time point. At each time point, the difference in gene expression was analyzed by comparing the gene expression level under ST with that under the corresponding control, not with that at the start point, which excluded the interference from genes whose expression levels changed regardless of the circumstances and general genetic backgrounds.

Analysis of the root transcriptome under salt-stress and control conditions, using a high-throughput sequencing method, identified the global gene-expression profiles occurring during salt-crop interactions. We used the DGE technology on *B. napus* roots under ST and control conditions and mapped the clean reads of *B. napus* to the genome of *B. rapa*. Based on the high collinearity and relationship of *B. rapa* and *B. napus* [36], over 30,000 *B. rapa* genes (41,019 unique genes) were matched to each library. Although about 30% of the total reads from each library were not mapped, saturation analysis revealed that the mapped genes were saturated, which indicated that the number of mapped genes would not increase with ADDITIONAL sequence data. More than 70% (30,000/41,019) of *B. rapa* genes were mapped, which was bigger than the percentages in poplar, *Reaumuria trigyna* and soybean under salt stress [38–40], showing that we obtained expression information of a sufficient number of genes and established a good foundation for subsequent analysis.

### 4.3 Candidate genes for salt tolerance in *B. napus*

Only 163 DEGs were identified as specifically responding to salt stress. The number of genes was fewer than that in other studies probably because we conducted “two-step filtering”: (1) Gene expression-levels were compared with their own time-point controls. (2) Taking intersections of the DEGs at three time points identified the common DEGs. We paid more attention to the DEGs identified throughout the time course instead of those gens which were changed at a single time point. Some of the DEGs identified in our study were also found by Liang *et al*. [17], such as the glycine-rich protein, ERD family proteins, glycosyl transferase family and ubiquitin-protein ligase. Many salt-responsive orthologous genes shared conserved functional roles across different plant species [41]. Similarly, some classical salt-related genes and metabolisms were confirmed in this study. Meanwhile, some new salt-responsive genes from our study may provide new clues to a deeper understanding of salt tolerance mechanisms in crops.

### 4.4 Genes related with the oxidation-reduction process respond to salt stress

Salt stress induces reactive oxygen species (ROS), which leads to secondary oxidation stress, disturbs cellular redox homeostasis, and damages cell components and structures [42]. Thus genes involved in the oxidation-reduction process may respond to salt stress. Through expression comparison, GO and KEGG enrichment analyses, some genes and related pathways were identified.

The proline metabolism pathway, an important pathway responding to oxidation stress, was represented by the most DEGs and was most significant during the stress course. The related enzyme-coding genes were in the top 10 list in our results (S2 Table). Of the genes involved in proline metabolism pathway, 4 genes were extremely down-regulated at 3 time points, and they were predicted to be encoded Pro dehydrogenase (ProDH), catalyzing proline to Δ^1^-Pyrroline-5-Carboxlyate (P5C), which were related with the core proline metabolism pathway [43]. In addition, although three up-regulated genes encoding the Δ^1^-pyrroline-5-carboxylate synthase (P5CS), which catalyzed the glutamate to P5C, were not in the top 10, the genes were common DEGs. Sustained down-regulation of *ProDH* and up-regulation of *P5CS* would make glutamate flow more towards proline, which agreed with the other studies [15–16].

Myo-inositol oxygenase (MIOX) may play an important role in alleviating over-production of ROS from salt stress. L-ascorbic acid (AsA) is an important component of the anti-oxidative system in plants, which provides crucial protection against oxidative damage induced by salt pressure [44]. In plants, there are two AsA biosynthetic pathways [45]. One is from D-gluctose-6-P to L-galactose, which is then oxidized by the NAD-dependent L-galactose dehydrogenase to form L-galactono-1,4-lactone, the immediate precursor of AsA. The other pathway is from myo-inositol (Ins) to L-galactono-1,4-lactone, and then to AsA. In the second pathway, MIOX catalyzes the first step, cleaving the Ins ring to form D-Glucuronic Acid (DGlcA). DGlcA is used to synthesize L-galactonic acid, which spontaneously converts to L-galactono-1,4-lactone and is oxidized to AsA. In this study, extreme differences in expressions levels of *MIOX* genes (especially for *MIOX4*) were observed during the stress process. Coincidentally, Lorence *et al*. [46] overexpressed the *MIOX4* gene in transgenic *Arabidopsis* and observed a 2- to 3-fold increase in AsA, suggesting a direct link between Ins oxidation and AsA synthesis. Combined with the results from GO and KEGG analyses, the *MIOX* genes may contribute to AsA synthesis. Although there have been some studies about the function of MIOX in AsA synthesis in recent years, none of the studies reveal the detailed function related to the response to salt stress [47–49]. Besides, expression variations of *MIOX* genes were proved to be involved in the changes of AsA in apple, pepper and strawberry [50–52], which provided some positive evidences for our hypothesis. In our results, the *MIOX* genes were consistently down-regulated in response to salt stress, which was also observed in rice roots in response to salinity stress [53]. The expression change may decrease AsA content. Because of the criterion of identifying differentially genes, only those genes which were differentially expressed at all the 3 time points were selected. Genes related to an alternative AsA synthesis pathway, a pathway that might compensate for the reduction in *MIOX*, were not found in the list of the 163 genes. There were no clues showed that this pathway was enhanced or inhibited under salt stress. However, enhancing the pathway from Ins to AsA by engineering *MIOX* genes may increase the antioxidant ability, which was probably a major step towards the development of salinity-tolerant crop plants [54].

Cytochrome P450s are hemethiolate enzymes participating in the redox reaction and are involved in numerous biosynthetic pathways in almost all organisms [55]. Two genes homologous with two P450s (*CYP71B37* and *CYP79B3*) were down-regulated under salt stress during the time course. *CYP79B3* is a critical enzyme for converting tryptophan to indole-3-acetaldoxime (IAOx), the intermediate in auxin biosynthesis *in vivo* [56]. Auxin is confirmed to participate in crosstalk with salt signals in plants [57, 58]. Besides, overexpression of *CYP79B3* could enhance root development [59]. *CYP71B37* was differentially expressed in response to drought and salinity in chickpea roots [60]. Therefore, we hypothesized that regulation of the expression of *CYP* genes may be linked with the inhibition of root growth under salt stress.

The UDP-Glycosyltransferases (UGTs) usually transfer UDP-xylose to low-molecular-weight aglycone substrates and alter their activity, solubility, and transport in plants. The substrates include multiple hormones (such as auxin, ABA, cytokinin, salicylic acid, and brassinosteroids), which alter ROS levels through regulation of ROS-scavenging related genes and hence modulate biotic and abiotic stress tolerance [61–62]. Langlois-Meurinne *et al*. [63] showed that expression of *UGT73B3* was necessary during the hypersensitive response. The expression variations in *UGT* expression were also observed in legume (http://bioinformatics.cau.edu.cn/MtED/probe_search.php?probeid=Mtr.44246.1.S1_at) and Citrus under salt stress [64]. In this study, two *UGT* homologous genes showed differential expression, suggesting that the *UGTs* may also participate in the salt stress response.

The 2-oxoglutarate and Fe(II)-dependent oxygenase (2OG oxygenase) couples two-electron oxidation of a substrate to the oxidative decarboxylation of 2-oxoglutarate to give succinate and carbon dioxide. A *DMR6* gene, encoding a 2-oxoglutarate (2OG)-Fe(II) oxygenase, mediates resistance to downy mildew by activating other defense genes [65]. The 2OG oxygenase modulates rice leaf rolling by affecting secondary cell wall formation in leaves, which protects plants from water stress [66]. The strong differential regulation of the 2OG oxygenase gene was also seen in the transcriptome of citrus under bacterial infection [64]. All these results suggested the function of the 2OG oxygenase in stress resistance. In our results, the genes encoding the 2OG oxygenase showed continuous down-regulation during the time course of salt stress, which implied a role of them in resistance to salt stress.

### 4.5 Genes in carbohydrate metabolic process function in salt stress

In this study, 6 genes encoding xyloglucan endotransglucosylase/hydrolases (XTHs) were found to be differentially expressed under salt stress. XTHs are proved in both the splitting and/or reconnection of xyloglucan cross-links, and are considered to play a critical role in both the construction and the disassembly of cell wall architecture, which can alter the compositions of cell walls by regulating their expression levels [67–70]. Cell wall, as a physical barrier surrounding the plant cell, is intimately involved in plant responses to the external environments through changes in its architecture and composition. And some studies reveal that salt stress affects cell walls severely, including the rigidity and composition [71].

Overexpression of *XTH* genes from *Populus euphratica* (a salt-tolerant tress species) in tobacco could increase salt tolerance by the development of leaf succulence via highly packed palisade parenchyma cells [72]. Constitutive expression of abiotic stress-inducible hot pepper *CaXTH3* improves drought and salt tolerance in transgenic *Arabidopsis* plants [73]. Many studies suggest *XTH* genes are related with plant tolerance to salt, though the molecular mechanisms remain unclear. The 6 *XTH* genes identified in our study could be important candidate genes for further study.

### 4.6 Possible mechanisms for the decrease of root length under salt stress

**Fig 10:**
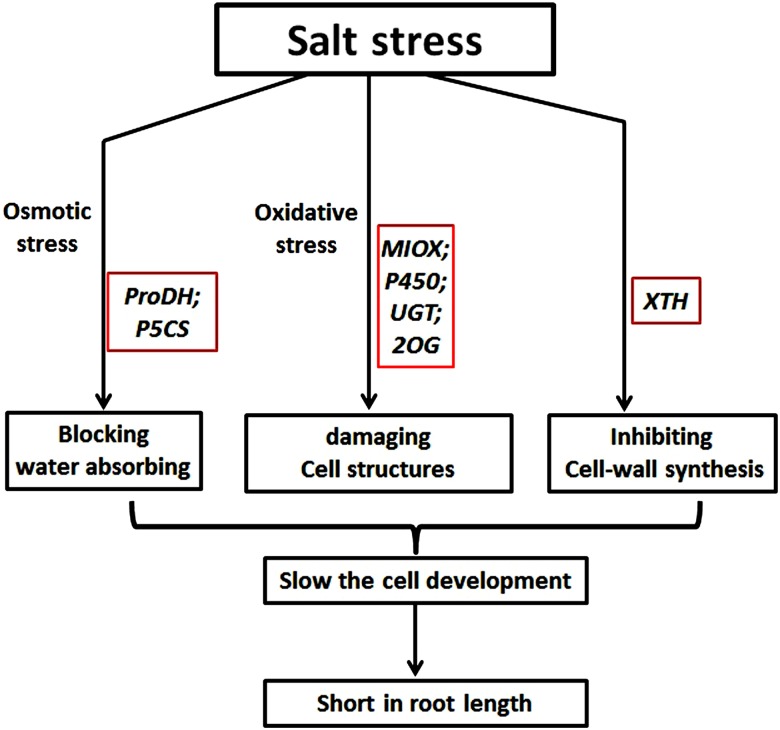
Proposed model for root-development inhibition under salt stress. Salt stress caused osmotic stress, oxidative stress and cell wall damage to the roots. Some salt-stress related genes (in red boxes) were regulated by, or responded to, each of these three aspects.

In this study, root length under control treatment was significantly longer than that under ST, which showed that salt stress inhibited root development. This implied that salt stress inhibited cell proliferation in the roots. Our results suggested some putative mechanisms to explain the shortened roots caused by salt stress: (1) High concentrations of NaCl in the environment decreased the water potential, which made it difficult for the plant cells to absorb external water [2]. The up-regulation of *P5CS* and down-regulation of *ProDH* increased proline synthesis, which could balance the water potential inside and outside the cells to some extent. This change would only inhibit the water flow from the cells to the environment and could not help cells absorb enough water to perform related substrate synthesis for cell development. Therefore, root development slowed down. (2) Large amounts of ROS were generated in root cells under ST, which may cause oxidative damage to cells [48]. Under control conditions, the ROS scavenging system may neutralize the excess ROS. By contrast, down-regulation of related genes (such as *MIOX* and P450) decreased antioxidant synthesis, which weakened the resistance to ROS under ST. Moreover, high amount of ROS damaged the existing active cells, which had a negative effect on cell proliferation. (3) The cell wall is the outermost structure of plant cells; therefore, salt stress must have some interactions with the cell wall and its constituent proteins. Cell wall loosening and extensibility, which occur during cell growth and division, are regulated by the *XTHs*. Down-regulation of *XTHs* under salt stress would negatively affect cell wall reconstruction and resynthesis, which would interfere with roots cell expansion and division. Taken together, salt stress inhibited root development in different ways at the germination stage, including by osmotic stress, oxidative stress and damage to the cell wall (Fig. 10).

## Conclusions

In summary, the root transcriptomes of *B. napus* at four time points under control and salt-stress conditions were explored using the Illumina sequencing platform. Approximately 1,500 genes were identified as DEGs after mapping the sequence data to the *B. rapa* genome. By comparing gene expression levels between salt treatment and the control at each time point, 163 genes were identified to be differentially expressed in response to salt stress throughout the time course, indicating that these genes functioned principally in *B. napus* under salt stress. Some important genes were identified by GO and KEGG analyses, which may play important roles in tolerance to the osmotic stress, oxidative stress and cell wall damage caused by salt stress. Thus, this study identified salt responding genes and their related metabolic pathways, and provided a platform for discovering salt tolerance mechanisms in plants. Manipulation of the candidate genes from this experiment may enhance crop salt tolerance.

## Supporting Information

S1 Fig Saturation analyses of seven libraries. (TIF)

S1 Table Primers of selected genes in Qpcr. (XLSX)

S2 Table Expression data of the 163 DEGs at each time point and their function analyses. (XLSX)

S3 Table Original data of qPCR for selected genes. (XLSX)
