# Supplementary figures and images for: Transcriptome Analysis of Canola (Brassica napus) under Salt Stress at the Germination Stage
Source: PLoS One. 2015 Feb 13;10(2):e0116217. doi: 10.1371/journal.pone.0116217 (PMC4332669; doi:10.1371/journal.pone.0116217)

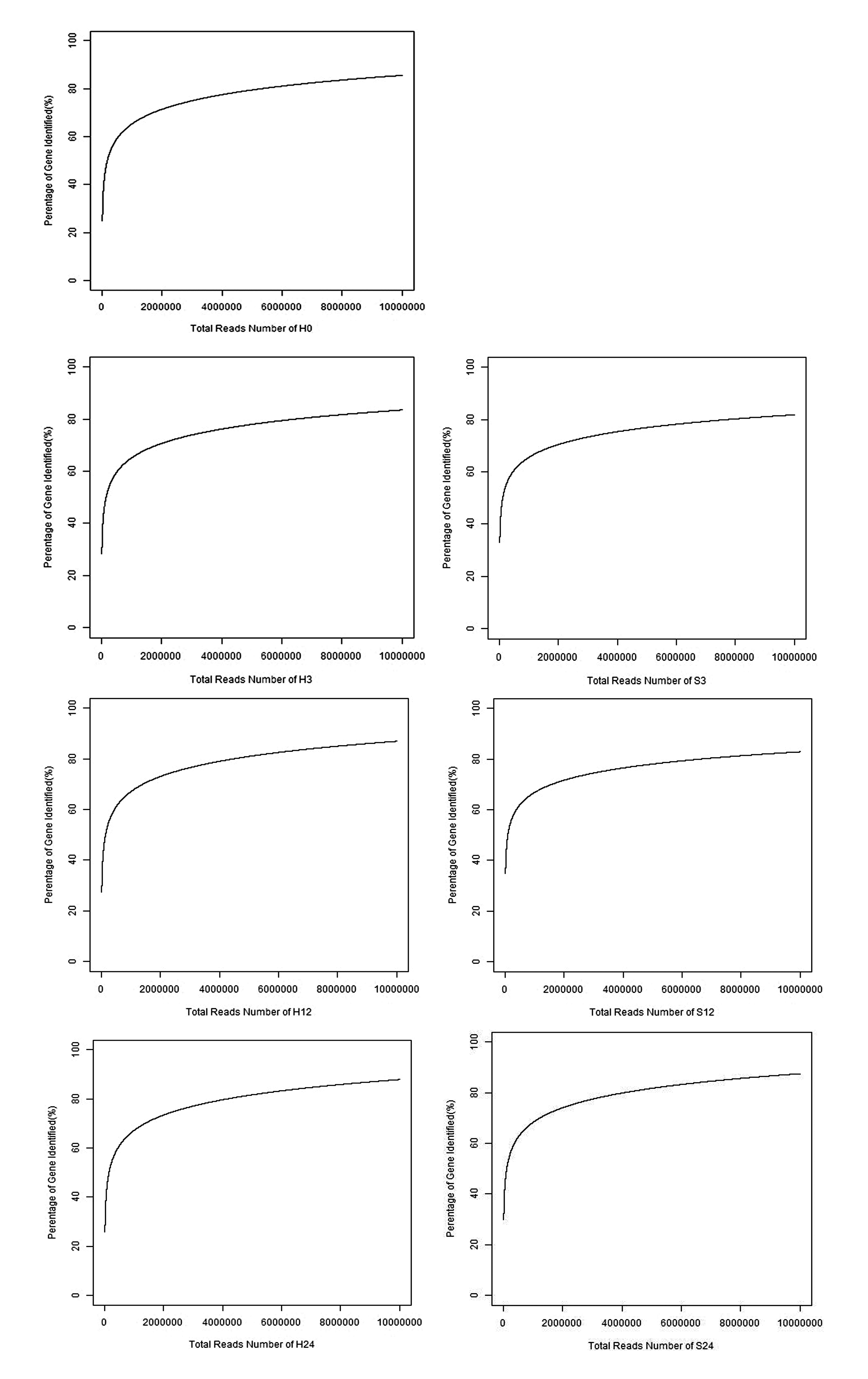

Supplement: S1 Fig — (TIF) [file pone.0116217.s001.tif]
